# Supplementary material for: A pre-post evaluation study of a social media-based COVID-19 communication campaign to improve attitudes and behaviors toward COVID-19 vaccination in Tanzania
Source: PLoS One. 2024 May 6;19(5):e0300206. doi: 10.1371/journal.pone.0300206 (PMC11073716; doi:10.1371/journal.pone.0300206)
Supplement: S1 File — (DOCX) [file pone.0300206.s001.docx]

A selected list of the media coverage of the One by One: Target COVID-19 pilot campaign in Tanzania

<https://www.rockefellerfoundation.org/news/africa-covid-champions-in-drc-zambia-and-uganda/>

<https://healthpolicy-watch.news/unitehealth-awards-to-highlight-power-of-social-media-networks-for-good-during-covid-19/>

<https://www.africa.com/africa-cdc-african-union-who-the-rockefeller-foundation-join-the-one-by-one-target-covid-19-campaign-to-launch-africacovidchampions-and-for-a-virtual-workshop/>

Social media coverage of the One by One: Target COVID-19 pilot campaign in Tanzania

(related hashtags include #AfricaCOVIDChampions #Tanzaniaikotayari #UjanjaKuchanja)

<https://twitter.com/hashtag/tanzaniaikotayri?src=hashtag_click>

<https://www.youtube.com/watch?v=YyyhWSBoYAU>

<https://www.instagram.com/explore/tags/tanzaniaikotayari/?img_index=1>

<https://www.instagram.com/onebyone2030/reel/Cg4SdARviCo/>
