# Supplementary material for: A pre-post evaluation study of a social media-based COVID-19 communication campaign to improve attitudes and behaviors toward COVID-19 vaccination in Tanzania
Source: PLoS One. 2024 May 6;19(5):e0300206. doi: 10.1371/journal.pone.0300206 (PMC11073716; doi:10.1371/journal.pone.0300206)
Supplement: S2 File — (PDF) [file pone.0300206.s002.pdf]

New York University • Office of Research Compliance • Institutional Review Board
665 Broadway, Suite 804• New York, NY 10012-2331 • https://www.nyu.edu/research/resources-and-support-offices/getting-started-withyourresearch/human-subjects-research.html
(212) 998-4808 • fax: (212) 995-4304 • ask.humansubjects@nyu.edu

Checklist for Determining Whether a Project Involves Human Subjects Research

Version: 1.3 Date: 03/06/2019

Please do not submit this form to the IRB Office. Please keep a copy with your research protocol.

This application does not replace a submission to determine if research is exempt from Federal human subjects research regulations. *Exempt is a type of review which at NYU must be carried out by the IRB to be deemed a valid determination. NYU policy does not provide investigators with authority to make an independent determination that an activity meets one or more of the categories of exempt human subjects research. Investigators who intend to conduct research that might involve human subjects will need to submit a formal application to the IRB via Cayuse IRB for determination before the research can begin. Guidance is available on the NYU IRB website.*

I. Research Title:

II. Personnel

A. Principal Investigator

Name (Last, First)	Degree(s)	University Status/Title
Tozan, Yesim	PhD	Assistant Professor
Department<br>Global and Environmental Public Health / Public Health Policy and Management		College<br>School of Global Public Health
Phone Number<br>(212) 998-5809		E-mail Address<br>tozan@nyu.edu

B. Faculty Sponsor – required when PI is a student

Name (Last, First)	Degree(s)	University Status/Title
Department		College
Phone Number		E-mail Address

III. Activities Determined to be Not Human Subjects Research as defined by the Federal Regulations

Indicate whether any of the below describe the activities associated with your project.

☐ Course-related activities specifically designed for educational or teaching purposes; where data is collected from and about human subjects as part of a classroom exercise or assignment and will not be used, published or presented outside of the classroom.

☐ The activity is a case report involving the observation of a single patient whose novel condition or response to treatment was guided by the care provider's judgment regarding the best interest of the individual.

☐ An activity only uses publicly available data such as census data or labor statistics. Note: Investigators should contact the IRB if they are uncertain as to whether the data qualifies as "publicly available." Please enter the names and URLs of the data sets, which will be used for your research: .

☐ An activity involves research that is limited to death records, autopsy materials, or cadaver specimens (provided that the cadaveric tissues/cells are not used for clinical investigations).

☐ The activity involves information-gathering interviews where questions focus on things, products, or policies rather than people or their thoughts regarding themselves. Examples: canvassing librarians about their libraries' inter-library loan policies or periodical purchases or interviews with company engineers or managers about how a product is made.

☐ The activity involves scholarly and journalistic research (*e.g.,* oral history, journalism, biography, literary criticism, legal research, and historical scholarship), including the collection and use of information, that focus directly on the specific individuals about whom the information is collected.

*If you checked any of the above boxes – STOP your activity is in a category that has been determined to **not** represent* human *subjects research. The requirement to apply human subjects research has been waived. You can skip to page 3, Section V, sign and retain this form in your files to document this determination. The IRB strongly recommends that investigators document this determination by placing a copy of this completed application in their files to address any future queries about the project.*

Note that the IRB does not provide formal determination letters based on the completion of this form. All investigators for whom a formal letter from the IRB is required must apply to Cayuse IRB.

If your proposed research does not fit any of the activities listed above, complete the remainder of this application or contact the IRB Office to determine if you must complete an application for IRB review.

IV. Criteria for Research Involving Human Subjects

A. Does the activity meet the definition of research?

Answer yes or no to the following:

- The activity employs a systematic approach involving predetermined methods for studying a specific topic, answering a specific question, testing a specific hypothesis, or developing a theory.
  ☒ Yes ☐ No

- The activity is intended to contribute to generalizable knowledge by extending the results beyond a single individual or an internal unit (e.g., publications or presentations).
  ☒ Yes ☐ No

B. Does the activity involve human subjects according to the definition?

Answer yes or no to the following:

- The investigator obtains specimens or data through intervention or interaction with a living individual (e.g., interviews, surveys, physical procedures, manipulations of the subject's environment, private or

limited access internet sites, or any other direct contact or communication with a subject).
☐ Yes ☒ No

- The investigator is obtaining identifiable private information about living individuals (e.g., chart reviews, lab studies on tissues or specimens, information from data or tissue repositories).
☐ Yes ☒ No

- The data or specimens are received by or provided to the investigator with identifiable private information.
☐ Yes ☒ No

- The data or specimens are coded, and the investigator has access to a link that would allow the data or samples to be identified.
☐ Yes ☒ No

Note: If you answered "yes" to both questions in Section IV.A **AND** "yes" to at least one question in Section IV.B. **STOP.** Your activity meets the definition of human subjects research, and either exempt, exempt limited IRB review, expedited or convened review is required.
For all projects that do NOT meet the definition of human subjects research, obtain appropriate signatures in Section V below and retain for your files.

For additional information regarding the review of human subjects research, please refer to the NYU IRB website.

V. SIGNATURES

Signature of Principal Investigator: _[signature]_ **Date** 02/26/2023

Signature of Faculty Sponsor:________ **Date**________
