# Supplementary material for: A pre-post evaluation study of a social media-based COVID-19 communication campaign to improve attitudes and behaviors toward COVID-19 vaccination in Tanzania
Source: PLoS One. 2024 May 6;19(5):e0300206. doi: 10.1371/journal.pone.0300206 (PMC11073716; doi:10.1371/journal.pone.0300206)
Supplement: S3 File — (DOCX) [file pone.0300206.s003.docx]

**Table S3-1. Survey questionnaire**

| # | Question | Options | Swahili Translation | Options |
| --- | --- | --- | --- | --- |
|  | Basic / Demographics | | |  |
| 1 | Please choose which language you would like to take the survey in. | English / Kiingereza  Swahili / Kiswahili | Chagua lugha ambayo ungependa kutumia kwenye utafiti huu. |  |
| 2 | What is your gender? | Male  Female | Jinsia? | Me  Ke |
| 3 | What is your age? | 18-24  25-34  35-44  45-54  55-64  65 and over | Umri? | 18-24  25-34  35-44  45-54  55-64  65 au zaidi |
| 4 | Which region do you currently reside in? | (Choose from drop-down menu) 31 regions | Mkoa unaoishi kwa sasa? |  |
| 5 | What is your work/employment status?  Please select all that apply. | Self-employed  Employed full-time  Employed part-time  Unemployed  Student  Retired  Housekeeper | Kazi?  Chagua majibu yote yaliyo sahihi kwako. | Nimejiajiri  Nina ajira ya kudumu  Nina ajira ya muda  Sina ajira  Mwanafunzi  Mstaafu  Msaidizi wa Nyumbani |
| 6 | What is the highest level of education you obtained? | No qualification  Primary school  Secondary School  A-Level Secondary School  Diploma  University degree or higher | Kiwango cha juu cha elimu? | Sijasoma kabisa  Elimu ya shule ya msingi  Elimu ya sekondari  Elimu ya kidato cha sita  Stashahada  Shahada au zaidi |
|  | Exposure status | | |  |
| 7 | What is your main source of acquiring COVID-19 information? (Check all that apply) | Social Media (e.g., Facebook, Twitter)  Google search (Wikipedia and other non-government websites)  TV  Radio and Podcasts  Newspaper (printed or internet)  Spouse/Partner  Other Family Members  Friends and Coworkers  Religious Leaders  Doctor/Medical Providers  Government websites  Official websites (e.g., CDC, WHO)  Other | Vyanzo vyako vikuu vya taarifa juu ya UVIKO 19 ni vipi?  Chagua vyanzo vikuu vitatu. | Mitandao ya kijamii (mf. Facebook, Twitter)  Google; Wikipedia na tovuti nyingine zisizo za kiserikali  Televisheni  Redio  Magazeti (Yaliyochapishwa au ya mtandaoni)  Mwenzi  Wanafamilia wengine  Marafiki na wafanyakazi wenza  Viongozi wa dini  Madaktari/watoa huduma za afya  Tovuti za serikali  Tovuti nyingine rasmi (mf. CDC, WHO)  Vyanzo vingine |
|  | Vaccination status (probing questions) | | |  |
| 10 | What is your COVID-19 vaccination status? | I am fully vaccinated  I am partially vaccinated  I am not vaccinated | Umeshapata chanjo ya UVIKO 19? | Nimepata chanjo kamili  Nimepata chanjo ya kwanza pekee  Bado sijachanja |
| 11 | Do you have an appointment to receive the remaining dose(s) of the vaccine? | Yes  No | Je una mpango wa kwenda kuchanja au kumalizia dozi iliyobaki? | Ndio  Hapana |
| Vaccine hesitancy scale  (only administered to those who are not fully vaccinated and have no vaccine appointment scheduled) | | | | |
| 12 | (If answered no to the above question) Would you get a government-approved COVID-19 vaccine if offered? | Definitely  Probably  I may or may not  Probably not  Definitely not  Don't know | Je, utakuwa tayari kupokea chanjo ya UVIKO19 iliyopitishwa na serikali? | Ndio  Inawezekana  Ninaweza au kuchanja au nisichanje  Haiwezekani  Hapana  Sijui |
| 13 | If there is a COVID-19 vaccine available | I will want to get it as soon as possible  I will take it when offered  I'm not sure what I will do  I will put off (delay) getting it  I will refuse to get it  Don't know | Kama chanjo ikiwepo: | Nitachanja haraka iwezekanavyo  Nitachanja nikipatiwa  Sina hakika nitafanya nini  Nitajichelewesha kuchanja  Nitakataa kuchanja  Sijui |
| 14 | I would describe my attitude towards receiving a COVID-19 vaccine as: | Very keen  Pretty positive  Neutral  Quite uneasy  Against it  Don't know | Mtazamo wangu juu ya chanjo ya UVIKO 19: | Niko makini sana  Mtazamo chanya  Sina mtazamo wowote  Nina wasiwasi nayo  Sikubaliani nayo  Sijui |
| 15 | If a COVID-19 vaccine was available at my local Health Facility, I would: | Get it as soon as possible  Get it when I have time  Delay getting it  Avoid getting it for as long as possible  Never get it  Don't know | Kama chanjo ya UVIKO 19 ingepatikana katika kituo cha afya cha karibu na mimi: | Ningechanja haraka iwezekanavyo  Ningechanja nikipata muda  Ningechelewa kuchanja  Ningekwepa kuchanja kadri iwezekanvyo  Nisingechanja kabisa  Sijui |
| 16 | If my family or friends were thinking of getting a COVID-19 vaccination, I would: | Strongly encourage them  Encourage them  Not say anything to them about it  Ask them to delay getting the vaccination  Suggest that they do not get the vaccination  Don't know | Kama familia yangu au marafiki wanafikiria kuhusu kuchanja chanjo ya UVIKO 19: | Ningewahimiza sana kuchanja  Ningewahimiza kuchanja  Nisingesema chochote kwao kuhusu chanjo  Ningewasihi wachelewe kuchanja  Ningewashauri wasichanje  Sijui |
| 17 | I would describe myself as: | Eager to get a COVID-19 vaccine  Willing to get the COVID-19 vaccine  Not bothered about getting the COVID-19 vaccine  Unwilling to get the COVID-19 vaccine  Anti-vaccination for COVID-19  Don't know | Ninaweza kujielezea kama mtu ambaye: | Ana shauku ya kupata chanjo ya UVIKO 19  Yuko tayari kupata chanjo ya UVIKO 19  Sisumbuliwi na suala la kupata chanjo ya UVIKO 19  Siko tayari kupata chanjo ya UVIKO 19  Nipo kinyume na chanjo ya UVIKO 19  Sijui |
| 18 | Taking a COVID-19 vaccination is: | Really important  Important  Neither important nor unimportant  Unimportant  Really unimportant  Don't know | Kuchanja chanjo ya UVIKO 19 ni: | Muhimu sana  Muhimu  Haina maana yoyote  Hakuna umuhimu  Hakuna umuhimu kabisa  Sijui |
| Vaccine confidence scale | | | | |
| 19 | If I get the COVID-19 vaccine it will be: | Really helpful for the community around me  Helpful for the community around me  Neither helpful nor unhelpful for the community around me  Unhelpful for the community around me  Really unhelpful for the community around me  Don’t know | Endapo nikipata chanjo ya UVIKO 19 itakuwa: | Msaada kubwa sana kwa jamii iliyonizunguka  Msaada kubwa kwa jamii iliyonizunguka  Haina maana yoyote kwa jamii iliyonizunguka  Haitokuwa na msaada kwa jamii iliyonizunguka  Haitokuwa na msaada kabisa kwa jamii iliyonizunguka  Sijui |
| 20 | If individuals like me get the COVID-19 vaccine it will: | Save a large number of lives  Save some lives  Have no impact  Lead to more deaths  Lead to a large number of deaths  Don’t know | Watu wengine kama mimi wakipata chanjo ya UVIKO 19: | Itaokoa maisha ya watu wengi sana  Itaokoa maisha ya watu kadhaa  Haitokuwa na athari yoyote  Itasababisha vifo zaidi  Itasababisha idadi kubwa zaidi ya vifo  Sijui |
| 21 | If many people do not get the vaccine this: | Will be dangerous  May be dangerous  Will have no consequences at all  May be good  Will be good  Don’t know | Kama watu wengi hawatochanja: | Itakuwa hatari  Inaweza ikawa hatari  Haitokuwa na matokeo yoyote  Pengine litakuwa jambo zuri  Litakuwa jambo zuri  Sijui |
| 22 | The COVID-19 vaccine will: | Greatly strengthen my immune system  Strengthen my immune system  It will neither strengthen nor weaken my immune system  Weaken my immune system  Greatly weaken my immune system  Don’t know | Chanjo ya UVIKO 19: | Itaimarisha kwa kiasi kikubwa sana mfumo wangu wa kinga  Itaimarisha mfumo wangu wa kinga  Haitoimarisha wala kudhoofisha mfumo wangu wa kinga  Itadhoofisha mfumo wangu wa kinga  Itadhoofisha sana mfumo wangu wa kinga  Sijui |
| 23 | Taking the COVID-19 vaccine: | Will give me complete freedom to get on with life just as before  Will give me greater freedom  Will have no effect on my freedom  Will restrict my freedom  Will completely restrict my freedom to get on with life  Don’t know | Kuchanja chanjo ya UVIKO 19; | Itanipa uhuru kamili wa kuendelea na maisha yangu kama hapo awali  Itanipa uhuru mkubwa zaidi  Haitokuwa na madhara yoyote juu ya uhuru wangu  Itazuia uhuru wangu  Itazuia kabisa uhuru wangu wa kuendelea na maisha  Sijui |
