# Supplementary material for: A pre-post evaluation study of a social media-based COVID-19 communication campaign to improve attitudes and behaviors toward COVID-19 vaccination in Tanzania
Source: PLoS One. 2024 May 6;19(5):e0300206. doi: 10.1371/journal.pone.0300206 (PMC11073716; doi:10.1371/journal.pone.0300206)
Supplement: S5 File — (DOCX) [file pone.0300206.s005.docx]

**Table S5-1. Overall sample characteristics (pre vs. post campaign)**

|  | **Pre-campaign (N=3,775)** | **Post-campaign (N=2,694)** | **Overall (N=6,469)** |  | ***p-value*** |
| --- | --- | --- | --- | --- | --- |
| **Sex** |  |  |  |  | <0.001 |
| Female | 461 (12.2%) | 397 (14.7%) | 858 (13.3%) |  |  |
| Male | 3,085 (81.7%) | 2,033 (75.5%) | 5,118 (79.1%) |  |  |
| Missing | 229 (6.1%) | 264 (9.8%) | 493 (7.6%) |  |  |
| **Age group** |  |  |  |  | <0.001 |
| 18-24 | 954 (25.3%) | 785 (29.1%) | 1739 (26.9%) |  |  |
| 25-34 | 2,177 (57.7%) | 1,489 (55.3%) | 3,666 (56.7%) |  |  |
| 35-44 | 329 (8.7%) | 125 (4.6%) | 454 (7.0%) |  |  |
| 45-54 | 61 (1.6%) | 15 (0.6%) | 76 (1.2%) |  |  |
| 55-64 | 9 (0.2%) | 7 (0.3%) | 16 (0.2%) |  |  |
| 65 and over | 5 (0.1%) | 4 (0.1%) | 9 (0.1%) |  |  |
| Missing | 240 (6.4%) | 269 (10.0%) | 509 (7.9%) |  |  |
| **Region of residence** |  |  |  |  | 0.639 |
| Dar es salaam region | 1,526 (40.4%) | 1,035 (38.4%) | 2,561 (39.6%) |  |  |
| Dodoma region | 229 (6.1%) | 183 (6.8%) | 412 (6.4%) |  |  |
| Arusha region | 235 (6.2%) | 154 (5.7%) | 389 (6.0%) |  |  |
| Mwanza region | 189 (5.0%) | 135 (5.0%) | 324 (5.0%) |  |  |
| Mbeya region | 165 (4.4%) | 97 (3.6%) | 262 (4.1%) |  |  |
| Kilimanjaro region | 116 (3.1%) | 79 (2.9%) | 195 (3.0%) |  |  |
| Other regions | 1,059 (28.1%) | 734 (27.2%) | 1,793 (27.7%) |  |  |
| Missing | 256 (6.8%) | 277 (10.3%) | 533 (8.2%) |  |  |
| **Occupation** |  |  |  |  | 0.030 |
| Employed full-time | 518 (13.7%) | 283 (10.5%) | 801 (12.4%) |  |  |
| Employed part-time | 645 (17.1%) | 493 (18.3%) | 1,138 (17.6%) |  |  |
| Housekeeper | 13 (0.3%) | 9 (0.3%) | 22 (0.3%) |  |  |
| Retired | 5 (0.1%) | 4 (0.1%) | 9 (0.1%) |  |  |
| Self-employed | 1,116 (29.6%) | 776 (28.8%) | 1,892 (29.2%) |  |  |
| Student | 501 (13.3%) | 329 (12.2%) | 830 (12.8%) |  |  |
| Unemployed | 713 (18.9%) | 513 (19.0%) | 1,226 (19.0%) |  |  |
| Missing | 264 (7.0%) | 287 (10.7%) | 551 (8.5%) |  |  |
| **Educational attainment** |  |  |  |  | <0.001 |
| No qualification | 11 (0.3%) | 10 (0.4%) | 21 (0.3%) |  |  |
| Primary school | 129 (3.4%) | 112 (4.2%) | 241 (3.7%) |  |  |
| Secondary school | 712 (18.9%) | 699 (25.9%) | 1,411 (21.8%) |  |  |
| A-level secondary school | 268 (7.1%) | 154 (5.7%) | 422 (6.5%) |  |  |
| Diploma | 894 (23.7%) | 650 (24.1%) | 1,544 (23.9%) |  |  |
| University degree or higher | 1,491 (39.5%) | 777 (28.8%) | 2,268 (35.1%) |  |  |
| Missing | 270 (7.2%) | 292 (10.8%) | 562 (8.7%) |  |  |
| **Campaign exposure status** |  |  |  |  | 0.979 |
| 0 | 569 (15.1%) | 389 (14.4%) | 958 (14.8%) |  |  |
| 1 | 2,873 (76.1%) | 1,973 (73.2%) | 4,846 (74.9%) |  |  |
| Missing | 333 (8.8%) | 332 (12.3%) | 665 (10.3%) |  |  |
| **Vaccine confidence score (Range 0-25)** |  |  |  |  | 0.001 |
| Mean (SD) | 22.4 (3.89) | 23.0 (3.32) | 22.7 (3.67) |  |  |
| Median [Min, Max] | 24.0 [0, 25.0] | 24.0 [0, 25.0] | 24.0 [0, 25.0] |  |  |
| Missing | 857 (22.7%) | 599 (22.2%) | 1,456 (22.5%) |  |  |
| **Vaccine hesitancy score (Range 0-35)** |  |  |  |  | 0.419 |
| Mean (SD) | 21.6 (6.86) | 22.3 (7.52) | 21.9 (7.12) |  |  |
| Median [Min, Max] | 23.0 [2.00, 34.0] | 24.0 [0, 34.0] | 23.0 [0, 34.0] |  |  |
| Missing | 3,061 (81.1%) | 2,268 (84.2%) | 5,329 (82.4%) |  |  |
| **Vaccination status** |  |  |  |  | <0.001 |
| 0 | 948 (25.1%) | 442 (16.4%) | 1,390 (21.5%) |  |  |
| 1 | 1,285 (34.0%) | 1,032 (38.3%) | 2,317 (35.8%) |  |  |
| Missing | 1,542 (40.8%) | 1,220 (45.3%) | 2,762 (42.7%) |  |  |

**Table S5-2. Overall sample characteristics at baseline (treatment vs. control group)**

|  | **Control (N=569)** | **Treatment (N=2,873)** | **Overall (N=3,775)** | ***p-value*** |  |  |  |  |
| --- | --- | --- | --- | --- | --- | --- | --- | --- |
| **Sex** |  |  |  | 0.791 |  |  |  |  |
| Female | 76 (13.4%) | 369 (12.8%) | 461 (12.2%) |  |  |  |  |  |
| Male | 493 (86.6%) | 2,504 (87.2%) | 3,085 (81.7%) |  |  |  |  |  |
| Missing | 0 (0%) | 0 (0%) | 229 (6.1%) |  |  |  |  |  |
| **Age group** |  |  |  | 0.011 |  |  |  |  |
| 18-24 | 151 (26.5%) | 773 (26.9%) | 954 (25.3%) |  |  |  |  |  |
| 25-34 | 330 (58.0%) | 1,795 (62.5%) | 2,177 (57.7%) |  |  |  |  |  |
| 35-44 | 67 (11.8%) | 255 (8.9%) | 329 (8.7%) |  |  |  |  |  |
| 45-54 | 17 (3.0%) | 41 (1.4%) | 61 (1.6%) |  |  |  |  |  |
| 55-64 | 3 (0.5%) | 6 (0.2%) | 9 (0.2%) |  |  |  |  |  |
| 65 and over | 1 (0.2%) | 3 (0.1%) | 5 (0.1%) |  |  |  |  |  |
| Missing | 0 (0%) | 0 (0%) | 240 (6.4%) |  |  |  |  |  |
| **Region of residence** |  |  |  | 0.433 |  |  |  |  |
| Dar es salaam region | 231 (40.6%) | 1,262 (43.9%) | 1,526 (40.4%) |  |  |  |  |  |
| Dodoma region | 45 (7.9%) | 182 (6.3%) | 229 (6.1%) |  |  |  |  |  |
| Arusha region | 40 (7.0%) | 188 (6.5%) | 235 (6.2%) |  |  |  |  |  |
| Mwanza region | 31 (5.4%) | 152 (5.3%) | 189 (5.0%) |  |  |  |  |  |
| Mbeya region | 23 (4.0%) | 140 (4.9%) | 165 (4.4%) |  |  |  |  |  |
| Kilimanjaro region | 15 (2.6%) | 98 (3.4%) | 116 (3.1%) |  |  |  |  |  |
| Other regions | 184 (32.3%) | 851 (29.6%) | 1,059 (28.1%) |  |  |  |  |  |
| Missing | 0 (0%) | 0 (0%) | 256 (6.8%) |  |  |  |  |  |
| **Occupation** |  |  |  | 0.165 |  |  |  |  |
| Employed full-time | 101 (17.8%) | 409 (14.2%) | 518 (13.7%) |  |  |  |  |  |
| Employed part-time | 106 (18.6%) | 526 (18.3%) | 645 (17.1%) |  |  |  |  |  |
| Housekeeper | 2 (0.4%) | 10 (0.3%) | 13 (0.3%) |  |  |  |  |  |
| Retired | 2 (0.4%) | 3 (0.1%) | 5 (0.1%) |  |  |  |  |  |
| Self-employed | 160 (28.1%) | 925 (32.2%) | 1,116 (29.6%) |  |  |  |  |  |
| Student | 86 (15.1%) | 403 (14.0%) | 501 (13.3%) |  |  |  |  |  |
| Unemployed | 112 (19.7%) | 597 (20.8%) | 713 (18.9%) |  |  |  |  |  |
| Missing | 0 (0%) | 0 (0%) | 264 (7.0%) |  |  |  |  |  |
| **Educational attainment** |  |  |  | 0.877 |  |  |  |  |
| No qualification | 3 (0.5%) | 7 (0.2%) | 11 (0.3%) |  |  |  |  |  |
| Primary school | 20 (3.5%) | 103 (3.6%) | 129 (3.4%) |  |  |  |  |  |
| Secondary school | 113 (19.9%) | 579 (20.2%) | 712 (18.9%) |  |  |  |  |  |
| A-level secondary school | 44 (7.7%) | 217 (7.6%) | 268 (7.1%) |  |  |  |  |  |
| Diploma | 151 (26.5%) | 729 (25.4%) | 894 (23.7%) |  |  |  |  |  |
| University degree or higher | 238 (41.8%) | 1,238 (43.1%) | 1,491 (39.5%) |  |  |  |  |  |
| Missing | 0 (0%) | 0 (0%) | 270 (7.2%) |  |  |  |  |  |
| **Vaccine confidence score (Range 0-25)** |  |  |  |  |  |  |  |  |
| Mean (SD) | 22.8 (3.31) | 22.3 (3.99) | 22.4 (3.89) | 0.692 |  |  |  |  |
| Median [Min, Max] | 24.0 [5.00, 25.0] | 24.0 [0, 25.0] | 24.0 [0, 25.0] |  |  |  |  |  |
| Missing | 81 (14.2%) | 443 (15.4%) | 857 (22.7%) |  |  |  |  |  |
| **Vaccine hesitancy score (Range 0-35)** |  |  |  | 0.565 |  |  |  |  |
| Mean (SD) | 21.7 (7.31) | 21.6 (6.80) | 21.6 (6.86) |  |  |  |  |  |
| Median [Min, Max] | 23.5 [2.00, 34.0] | 23.0 [3.00, 34.0] | 23.0 [2.00, 34.0] |  |  |  |  |  |
| Missing | 479 (84.2%) | 2,249 (78.3%) | 3,061 (81.1%) |  |  |  |  |  |
| **Vaccination status** |  |  |  | <0.001 |  |  |  |  |
| Not fully vaccinated | 121 (21.3%) | 827 (28.8%) | 948 (25.1%) |  |  |  |  |  |
| Fully vaccinated + partially vaccinated with confirmed appointment(s) | 252 (44.3%) | 1,033 (36.0%) | 1,285 (34.0%) |  |  |  |  |  |
| Missing | 196 (34.4%) | 1,013 (35.3%) | 1,542 (40.8%) |  |  |  |  |  |

**Table S5-3. Overall sample characteristics at endline (treatment vs. control)**

|  | **Control (N=389)** | **Treatment (N=1,973)** | **Overall (N=2,694)** | ***p-value*** |  |  |
| --- | --- | --- | --- | --- | --- | --- |
| **Sex** |  |  |  | 0.698 |  |  |
| Female | 60 (15.4%) | 323 (16.4%) | 397 (14.7%) |  |  |  |
| Male | 329 (84.6%) | 1,650 (83.6%) | 2,033 (75.5%) |  |  |  |
| Missing | 0 (0%) | 0 (0%) | 264 (9.8%) |  |  |  |
| **Age group** |  |  |  | 0.002 |  |  |
| 18-24 | 105 (27.0%) | 656 (33.2%) | 785 (29.1%) |  |  |  |
| 25-34 | 253 (65.0%) | 1,203 (61.0%) | 1,489 (55.3%) |  |  |  |
| 35-44 | 21 (5.4%) | 101 (5.1%) | 125 (4.6%) |  |  |  |
| 45-54 | 5 (1.3%) | 8 (0.4%) | 15 (0.6%) |  |  |  |
| 55-64 | 3 (0.8%) | 4 (0.2%) | 7 (0.3%) |  |  |  |
| 65 and over | 2 (0.5%) | 1 (0.1%) | 4 (0.1%) |  |  |  |
| Missing | 0 (0%) | 0 (0%) | 269 (10.0%) |  |  |  |
| **Region of residence** |  |  |  | 0.853 |  |  |
| Dar es salaam region | 159 (40.9%) | 853 (43.2%) | 1,035 (38.4%) |  |  |  |
| Dodoma region | 32 (8.2%) | 149 (7.6%) | 183 (6.8%) |  |  |  |
| Arusha region | 31 (8.0%) | 120 (6.1%) | 154 (5.7%) |  |  |  |
| Mwanza region | 21 (5.4%) | 112 (5.7%) | 135 (5.0%) |  |  |  |
| Mbeya region | 16 (4.1%) | 78 (4.0%) | 97 (3.6%) |  |  |  |
| Kilimanjaro region | 14 (3.6%) | 64 (3.2%) | 79 (2.9%) |  |  |  |
| Other regions | 116 (29.8%) | 597 (30.3%) | 734 (27.2%) |  |  |  |
| Missing | 0 (0%) | 0 (0%) | 277 (10.3%) |  |  |  |
| **Occupation** |  |  |  | 0.028 |  |  |
| Employed full-time | 50 (12.9%) | 231 (11.7%) | 283 (10.5%) |  |  |  |
| Employed part-time | 96 (24.7%) | 388 (19.7%) | 493 (18.3%) |  |  |  |
| Housekeeper | 1 (0.3%) | 7 (0.4%) | 9 (0.3%) |  |  |  |
| Retired | 2 (0.5%) | 1 (0.1%) | 4 (0.1%) |  |  |  |
| Self-employed | 116 (29.8%) | 644 (32.6%) | 776 (28.8%) |  |  |  |
| Student | 41 (10.5%) | 283 (14.3%) | 329 (12.2%) |  |  |  |
| Unemployed | 83 (21.3%) | 419 (21.2%) | 513 (19.0%) |  |  |  |
| Missing | 0 (0%) | 0 (0%) | 287 (10.7%) |  |  |  |
| **Educational attainment** |  |  |  | 0.643 |  |  |
| No qualification | 3 (0.8%) | 5 (0.3%) | 10 (0.4%) |  |  |  |
| Primary school | 19 (4.9%) | 88 (4.5%) | 112 (4.2%) |  |  |  |
| Secondary school | 114 (29.3%) | 572 (29.0%) | 699 (25.9%) |  |  |  |
| A-level secondary school | 23 (5.9%) | 130 (6.6%) | 154 (5.7%) |  |  |  |
| Diploma | 109 (28.0%) | 531 (26.9%) | 650 (24.1%) |  |  |  |
| University degree or higher | 121 (31.1%) | 647 (32.8%) | 777 (28.8%) |  |  |  |
| Missing | 0 (0%) | 0 (0%) | 292 (10.8%) |  |  |  |
| **Vaccine confidence score (Range 0 - 25)** |  |  |  | 0.530 |  |  |
| Mean (SD) | 23.0 (3.68) | 23.0 (3.25) | 23.0 (3.32) |  |  |  |
| Median [Min, Max] | 24.0 [0, 25.0] | 24.0 [0, 25.0] | 24.0 [0, 25.0] |  |  |  |
| Missing | 47 (12.1%) | 220 (11.2%) | 599 (22.2%) |  |  |  |
| **Vaccine hesitancy score (Range 0 - 35)** |  |  |  | 0.752 |  |  |
| Mean (SD) | 22.9 (7.59) | 22.2 (7.52) | 22.3 (7.52) |  |  |  |
| Median [Min, Max] | 24.0 [0, 33.0] | 24.0 [0, 34.0] | 24.0 [0, 34.0] |  |  |  |
| Missing | 330 (84.8%) | 1,606 (81.4%) | 2,268 (84.2%) |  |  |  |
| **Vaccination status** |  |  |  | 0.069 |  |  |
| Not fully vaccinated | 65 (16.7%) | 377 (19.1%) | 442 (16.4%) |  |  |  |
| Fully vaccinated + partially vaccinated with confirmed appointment(s) | 194 (49.9%) | 838 (42.5%) | 1,032 (38.3%) |  |  |  |
| Missing | 130 (33.4%) | 758 (38.4%) | 1,220 (45.3%) |  |  |  |

**Table S5-4. Weighted sample characteristics using the propensity score-based matching proposed by Stuart et al.^1^ (treatment vs. control)**

| **Characteristic** | **Control**  **(N = 5,763)*^1^*** | **Treatment**  **(N = 5,756)*^1^*** |
| --- | --- | --- |
| **Age group** |  |  |
| 18-24 | 1,507 (26%) | 1,534 (27%) |
| 25-34 | 3,620 (63%) | 3,598 (63%) |
| 35-44 | 539 (9.4%) | 521 (9.0%) |
| 45-54 | 79 (1.4%) | 81 (1.4%) |
| 55-64 | 13 (0.2%) | 12 (0.2%) |
| 65 and over | 5 (<0.1%) | 10 (0.2%) |
| **Sex** |  |  |
| Female | 720 (12%) | 732 (13%) |
| Male | 5,043 (88%) | 5,024 (87%) |
| **Educational attainment** |  |  |
| No qualification | 14 (0.3%) | 14 (0.2%) |
| Primary school | 203 (3.5%) | 206 (3.6%) |
| Secondary school | 1,173 (20%) | 1,148 (20%) |
| A-level secondary school | 436 (7.6%) | 445 (7.7%) |
| Diploma | 1,439 (25%) | 1,461 (25%) |
| University degree or higher | 2,498 (43%) | 2,482 (43%) |
| **Occupation** |  |  |
| Employed full-time | 843 (15%) | 808 (14%) |
| Employed part-time | 1,037 (18%) | 1,043 (18%) |
| Housekeeper | 20 (0.3%) | 20 (0.4%) |
| Retired | 5 (<0.1%) | 10 (0.2%) |
| Self-employed | 1,890 (33%) | 1,868 (32%) |
| Student | 792 (14%) | 807 (14%) |
| Unemployed | 1,176 (20%) | 1,199 (21%) |
| **Pre/post** |  |  |
| pre | 2,873 (50%) | 2,873 (50%) |
| post | 2,890 (50%) | 2,883 (50%) |
| **Vaccine hesitancy score** | 22 (SD = 7) | 22 (SD = 7) |
| Missing | 4,897 | 4,609 |
| **Vaccine confidence score** | 22.9 (SD = 3.6) | 22.6 (SD = 3.7) |
| Missing | 711 | 744 |
| **Vaccination status** |  |  |
| Not fully vaccinated | 1,129 (30%) | 1,411 (38%) |
| Fully vaccinated + partially vaccinated with confirmed appointment(s) | 2,677 (70%) | 2,284 (62%) |
| Missing | 1,957 | 2,062 |
| ***^1^* n (%); Mean (SD = SD)** | | |

**
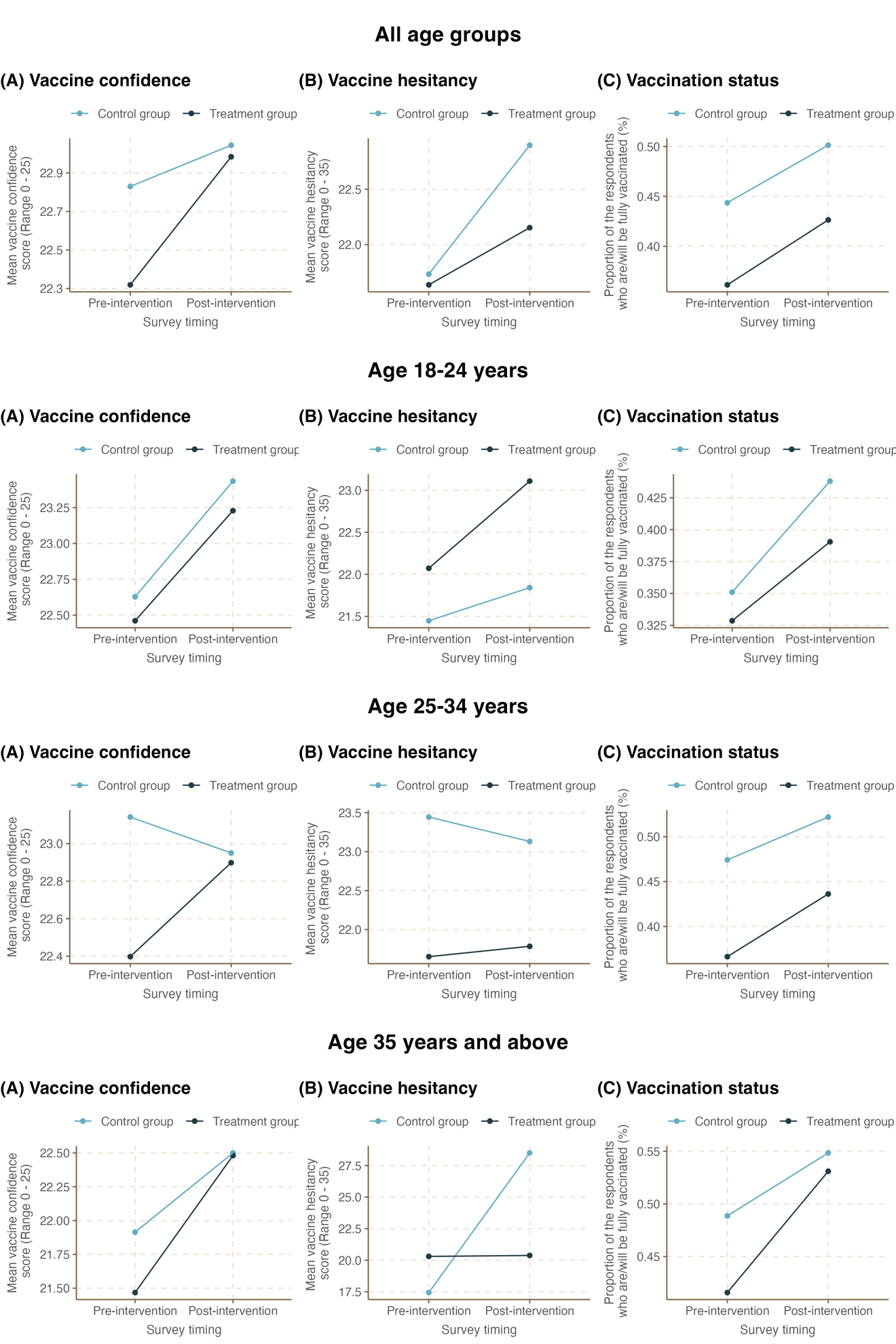
**

**Figure S5-1. Descriptive plot of changes in COVID-19 vaccine confidence, hesitancy, and vaccination status before and after the campaign among the campaign-exposed vs. non-exposed respondents stratified by age groups.**

**Table S5-5. The frequencies and percentages for individuals who chose each category in the Vaccine Confidence Scale**

|  | Overall (N=5804) |
| --- | --- |
| If I get the COVID-19 vaccine it will be: |  |
| Really helpful for the community around me | 3407 (58.7%) |
| Helpful for the community around me | 1449 (25.0%) |
| Neither helpful nor unhelpful for the community around me | 183 (3.2%) |
| Unhelpful for the community around me | 94 (1.6%) |
| Really unhelpful for the community around me | 70 (1.2%) |
| Don't know | 377 (6.5%) |
| Missing | 224 (3.9%) |
| If individuals like me get the COVID-19 vaccine it will: |  |
| Save a large number of lives | 4209 (72.5%) |
| Save some lives | 709 (12.2%) |
| Have no impact | 268 (4.6%) |
| Lead to more deaths | 31 (0.5%) |
| Lead to a large number of deaths | 22 (0.4%) |
| Don't know | 323 (5.6%) |
| Missing | 242 (4.2%) |
| If many people do not get the vaccine this: |  |
| Will be dangerous | 2969 (51.2%) |
| May be dangerous | 1910 (32.9%) |
| Will have no consequences at all | 220 (3.8%) |
| May be good | 149 (2.6%) |
| Will be good | 68 (1.2%) |
| Don't know | 240 (4.2%) |
| Missing | 248 (4.3%) |
| The COVID-19 vaccine will: |  |
| Greatly strengthen my immune system | 3191 (55.0%) |
| Strengthen my immune system | 1494 (25.7%) |
| It will neither strengthen nor weaken my immune system | 267 (4.6%) |
| Weaken my immune system | 78 (1.3%) |
| Greatly weaken my immune system | 52 (0.9%) |
| Don't know | 458 (7.9%) |
| Missing | 264 (4.5%) |
| Taking the COVID-19 vaccine: |  |
| Will give me complete freedom to get on with life just as before | 3411 (58.8%) |
| Will give me greater freedom | 1161 (20.0%) |
| Will have no effect on my freedom | 464 (8.0%) |
| Will restrict my freedom | 50 (0.9%) |
| Will completely restrict my freedom to get on with life | 52 (0.9%) |
| Don’t know | 385 (6.7%) |
| Missing | 281 (4.8%) |

**Table S5-6. The frequencies and percentages for individuals who chose each category in the Vaccine Hesitancy Scale (only prompted to the individuals who are not fully vaccinated or do not have appointment(s) to get fully vaccinated)**

|  | Overall (N=5804) |
| --- | --- |
| Would you get a government-approved COVID-19 vaccine if offered? |  |
| Definitely | 2349 (40.5%) |
| Probably | 526 (9.1%) |
| I may or may not | 659 (11.4%) |
| Probably not | 71 (1.2%) |
| Definitely not | 294 (5.1%) |
| Don't know | 168 (2.9%) |
| Missing | 1737 (29.9%) |
| If there is a COVID-19 vaccine available |  |
| I will want to get it as soon as possible | 1359 (25.1%) |
| I will take it when offered | 1338 (23.0%) |
| I'm not sure what i will do | 627 (10.8%) |
| I will put off (delay) getting it | 130 (2.3%) |
| I will refuse to get it | 227 (3.9%) |
| Don't know | 264 (4.5%) |
| Missing | 1759 (30.3%) |
| I would describe my attitude towards receiving a COVID-19 vaccine as |  |
| Very keen | 1117 (19.2%) |
| Pretty positive | 1728 (29.8%) |
| Neutral | 481 (8.3%) |
| Quite uneasy | 451 (7.8%) |
| Against it | 147 (2.5%) |
| Don't know | 103 (1.8%) |
| Missing | 1777 (30.6%) |
| If a COVID-19 vaccine was available at my local Health Facility, I would: |  |
| Get it as soon as possible | 1914 (33.0%) |
| Get it when I have time | 1168 (20.1%) |
| Delay getting it | 241 (4.2%) |
| Avoid getting it for as long as possible | 94 (1.6%) |
| Never get it | 266 (4.6%) |
| Don't know | 325 (5.6%) |
| Missing | 1796 (30.9%) |
| If my family or friends were thinking of getting a COVID-19 vaccination, I would: |  |
| Strongly encourage them | 1729 (29.8%) |
| Encourage them | 1366 (23.5%) |
| Not say anything to them about it | 522 (9.0%) |
| Ask them to delay getting the vaccination | 77 (1.3%) |
| Suggest that they do not get the vaccination | 114 (2.0%) |
| Don't know | 174 (3.0%) |
| Missing | 1822 (31.4%) |
| I would describe myself as: |  |
| Eager to get a covid-19 vaccine | 709 (12.2%) |
| Willing to get the covid-19 vaccine | 2208 (38.0%) |
| Not bothered about getting the covid-19 vaccine | 550 (9.5%) |
| Unwilling to get the covid-19 vaccine | 225 (3.9%) |
| Anti-vaccination for covid-19 | 90 (1.6%) |
| Don't know | 182 (3.1%) |
| Missing | 1840 (31.7%) |
| Taking a COVID-19 vaccination is: |  |
| Really important | 1953 (33.6%) |
| Important | 1369 (23.6%) |
| Neither important nor unimportant | 181 (3.1%) |
| Unimportant | 127 (2.2%) |
| Really unimportant | 73 (1.3%) |
| Don't know | 241 (4.2%) |
| Missing | 1860 (32.0%) |

**Table S5-7. Impact on COVID-19 vaccine hesitancy of the social-media based COVID-19 campaign in Tanzania**

|  | **Crude model**  **(N= 1,132)** | | | **Adjusted model***  **(N= 1,102)** | | | **Weighted/adjusted model**** | | |
| --- | --- | --- | --- | --- | --- | --- | --- | --- | --- |
| **Variables** | **Beta** | **95% CI***^1^* | **p-value** | **Beta** | **95% CI***^1^* | **p-value** | **Beta** | **95% CI***^1^* | **p-value** |
| **Prepost** | 1.2 | (-1.2, 3.5) | 0.329 | 0.51 | (-1.9, 2.9) | 0.673 | 0.55 | (-2.0, 3.1) | 0.670 |
| **Treatment** | -0.10 | (-1.7, 1.5) | **0.904** | -0.28 | (-1.9, 1.3) | 0.728 | -0.56 | (-2.1, 0.97) | 0.471 |
| **Prepost*Treatment** | -0.65 | (-3.2, 1.9) | 0.613 | -0.22 | (-2.7, 2.3) | 0.864 | -0.41 | (-3.1, 2.3) | 0.766 |
| * Model adjusted for age group, gender, highest educational attainment, and occupation  ** Treatment and control groups matched and adjusted by age group, ender, highest educational attainment, and occupation  *^1^* CI = Confidence Interval | | | | | | | | | |

**Table S5-8. Impact on COVID-19 vaccine confidence of the social media-based COVID-19 campaign in Tanzania**

|  | **Crude model**  **(n= 5005)** | | | **Adjusted model***  **(n= 4971)** | | | **Weighted/adjusted model**** | | |
| --- | --- | --- | --- | --- | --- | --- | --- | --- | --- |
| **Variables** | **Beta** | **95% CI***^1^* | **p-value** | **Beta** | **95% CI***^1^* | **p-value** | **Beta** | **95% CI***^1^* | **p-value** |
| **Prepost** | 0.21 | -0.29, 0.72 | 0.407 | 0.02 | -0.48, 0.52 | 0.931 | 0.02 | -0.52, 0.57 | 0.929 |
| **Treatment** | -0.51 | -0.87, -0.15 | 0.005 | -0.53 | -0.88, -0.18 | 0.003 | -0.05 | -0.87, -0.23 | <0.001 |
| **Prepost*Treatment** | 0.45 | -0.10, 1.0 | 0.111 | 0.47 | -0.07, 1.0 | 0.091 | 0.52 | -0.08, 1.1 | 0.087 |
| * Model adjusted for age group, gender, highest educational attainment, and occupation  ** Treatment and control groups matched and adjusted by age group, ender, highest educational attainment, and occupation  *^1^* CI = Confidence Interval | | | | | | | | | |

**Table S5-9. Impact on vaccination uptake of the social media-based COVID-19 campaign in Tanzania (including those respondents who had a confirmed appointment for remaining doses)**

|  | **Crude model**  **(n= 3699)** | | | **Adjusted model***  **(n= 3665)** | | | **Weighted/adjusted model**** | | |
| --- | --- | --- | --- | --- | --- | --- | --- | --- | --- |
| **Variables** | **Odds Ratio** | **95% CI***^1^* | **p-value** | **Odds Ratio** | **95% CI***^1^* | **p-value** | **Odds Ratio** | **95% CI***^1^* | **p-value** |
| **Prepost** | 1.43 | 1.01, 2.05 | 0.047 | 1.35 | 0.94, 1.95 | 0.107 | 1.07 | 0.99, 1.15 | 0.081 |
| **Treatment** | 0.60 | 0.47, 0.76 | <0.001 | 0.61 | 0.48, 0.78 | <0.001 | 0.89 | 0.85, 0.94 | <0.001 |
| **Prepost*Treatment** | 1.24 | 0.84, 1.82 | 0.272 | 1.25 | 0.84, 1.85 | 0.267 | 1.06 | 0.98, 1.15 | 0.156 |
| * Model adjusted for age group, gender, highest educational attainment, and occupation  ** Treatment and control groups matched and adjusted by age group, ender, highest educational attainment, and occupation  *^1^* CI = Confidence Interval | | | | | | | | | |

**Table S5-10. Impact of the campaign on the change in vaccine hesitancy among the respondents of age 18-24 years**

|  | **Crude model** | | | **Adjusted model*** | | | **Weighted/adjusted model**** | | |
| --- | --- | --- | --- | --- | --- | --- | --- | --- | --- |
| **Variables** | **Beta** | **95% CI***^1^* | **p-value** | **Beta** | **95% CI***^1^* | **p-value** | **Beta** | **95% CI***^1^* | **p-value** |
| **Prepost** | 0.39 | -3.8, 4.6 | 0.854 | 0.22 | -4.2, 4.6 | 0.924 | -1.2 | -6.9, 4.5 | 0.678 |
| **Treatment** | 0.62 | -2.2, 3.5 | 0.668 | 0.69 | -2.2, 3.6 | 0.641 | 1.2 | -1.9, 4.3 | 0.448 |
| **Prepost*Treatment** | 0.64 | -3.9, 5.2 | 0.781 | 0.58 | -4.1, 5.3 | 0.808 | 1.9 | -3.9, 7.7 | 0.520 |
| * Model adjusted for gender, highest educational attainment, and occupation  ** Treatment and control group matched and adjusted by age group, ender, highest educational attainment, and occupation  *^1^* CI = Confidence Interval | | | | | | | | | |

**Table S5-11. Impact of the campaign on the change in vaccine confidence among the respondents of age 18-24 years**

|  | **Crude model** | | | **Adjusted model*** | | | **Weighted/adjusted model**** | | |
| --- | --- | --- | --- | --- | --- | --- | --- | --- | --- |
| **Variables** | **Beta** | **95% CI***^1^* | **p-value** | **Beta** | **95% CI***^1^* | **p-value** | **Beta** | **95% CI***^1^* | **p-value** |
| **Prepost** | 0.81 | -0.09, 1.7 | 0.079 | 0.47 | -0.42, 1.4 | 0.303 | 0.78 | -0.24, 1.8 | 0.132 |
| **Treatment** | -0.17 | -0.80, 0.47 | 0.606 | -0.18 | -0.80, 0.45 | 0.579 | -0.19 | -0.76, 0.39 | 0.518 |
| **Prepost*Treatment** | -0.04 | -1.0, 0.94 | 0.937 | 0.12 | -0.85, 1.1 | 0.813 | -0.11 | -1.2, 0.99 | 0.847 |
| * Model adjusted for gender, highest educational attainment, and occupation  ** Treatment and control group matched and adjusted by age group, ender, highest educational attainment, and occupation  *^1^* CI = Confidence Interval | | | | | | | | | |

**Table S5-12. Impact of the campaign on the change in % of fully vaccinated (including the one who has a confirmed appointment for the remaining doses) among the respondents of age 18-24 years**

|  | **Crude model** | | | **Adjusted model*** | | | **Weighted/adjusted model**** | | |
| --- | --- | --- | --- | --- | --- | --- | --- | --- | --- |
| **Variables** | **Odds ratio** | **95% CI***^1^* | **p-value** | **Odds ratio** | **95% CI***^1^* | **p-value** | **Odds ratio** | **95% CI***^1^* | **p-value** |
| **Prepost** | 2.26 | 1.12, 4.71 | 0.026 | 1.86 | 0.90, 3.94 | 0.098 | 2.17 | 0.92, 5.09 | 0.057 |
| **Treatment** | 0.90 | 0.57, 1.40 | 0.631 | 0.83 | 0.52, 1.33 | 0.446 | 0.80 | 0.50, 1.27 | 0.327 |
| **Prepost*Treatment** | 0.77 | 0.35, 1.64 | 0.501 | 0.84 | 0.37, 1.82 | 0.659 | 0.70 | 0.28, 1.74 | 0.483 |
| * Model adjusted for gender, highest educational attainment, and occupation  ** Treatment and control group matched and adjusted by age group, ender, highest educational attainment, and occupation  *^1^* CI = Confidence Interval | | | | | | | | | |

**Table S5-13. Impact of the campaign on the change in vaccine hesitancy among the respondents of age 25-34 years**

|  | **Crude model** | | | **Adjusted model*** | | | **Weighted/adjusted model**** | | |
| --- | --- | --- | --- | --- | --- | --- | --- | --- | --- |
| **Variables** | **Beta** | **95% CI***^1^* | **p-value** | **Beta** | **95% CI***^1^* | **p-value** | **Beta** | **95% CI***^1^* | **p-value** |
| **Prepost** | -0.31 | -3.4, 2.7 | 0.841 | -0.81 | -3.9, 2.3 | 0.608 | -0.53 | -3.5, 2.4 | 0.721 |
| **Treatment** | -1.8 | -4.0, 0.39 | 0.108 | -2.1 | -4.3, 0.16 | 0.069 | -2.1 | -4.0, -0.10 | 0.039 |
| **Prepost*Treatment** | 0.45 | -2.8, 3.7 | 0.789 | 0.88 | -2.4, 4.2 | 0.601 | 0.61 | -2.5, 3.8 | 0.703 |
| * Model adjusted for gender, highest educational attainment, and occupation  ** Treatment and control group matched and adjusted by age group, ender, highest educational attainment, and occupation  *^1^* CI = Confidence Interval | | | | | | | | | |

**Table S5-14. Impact of the campaign on the change in vaccine confidence among the respondents of age 25-34 years**

|  | **Crude model** | | | **Adjusted model*** | | | **Weighted/adjusted model**** | | |
| --- | --- | --- | --- | --- | --- | --- | --- | --- | --- |
| **Variables** | **Beta** | **95% CI***^1^* | **p-value** | **Beta** | **95% CI***^1^* | **p-value** | **Beta** | **95% CI***^1^* | **p-value** |
| **Prepost** | -0.19 | -0.83, 0.44 | 0.555 | -0.26 | -0.89, 0.37 | 0.418 | -0.33 | -0.96, 0.31 | 0.313 |
| **Treatment** | -0.74 | -1.2, -0.29 | 0.001 | -0.73 | -1.2, -0.28 | 0.002 | -0.76 | -1.1, -0.37 | <0.001 |
| **Prepost*Treatment** | 0.69 | 0.00, 1.4 | 0.052 | 0.65 | -0.04, 1.3 | 0.063 | 0.76 | 0.06, 1.5 | 0.034 |
| * Model adjusted for gender, highest educational attainment, and occupation  ** Treatment and control group matched and adjusted by age group, ender, highest educational attainment, and occupation  *^1^* CI = Confidence Interval | | | | | | | | | |

**Table S5-15. Impact of the campaign on the change in % of fully vaccinated (including the one who has a confirmed appointment for the remaining doses) among the respondents of age 25-34 years**

|  | **Crude model** | | | **Adjusted model*** | | | **Weighted/adjusted model**** | | |
| --- | --- | --- | --- | --- | --- | --- | --- | --- | --- |
| **Variables** | **Odds ratio** | **95% CI***^1^* | **p-value** | **Odds ratio** | **95% CI***^1^* | **p-value** | **Odds ratio** | **95% CI***^1^* | **p-value** |
| **Prepost** | 1.05 | 0.67, 1.64 | 0.831 | 1.00 | 0.64, 1.58 | **0.999** | 0.99 | 0.62, 1.57 | 0.949 |
| **Treatment** | 0.49 | 0.35, 0.67 | <0.001 | 0.50 | 0.36, 0.69 | <0.001 | 0.51 | 0.37, 0.71 | <0.001 |
| **Prepost*Treatment** | 1.65 | 1.02, 2.68 | 0.042 | 1.64 | 1.00, 2.68 | 0.049 | 1.69 | 1.02, 2.81 | 0.023 |
| * Model adjusted for gender, highest educational attainment, and occupation  ** Treatment and control group matched and adjusted by age group, ender, highest educational attainment, and occupation  *^1^* CI = Confidence Interval | | | | | | | | | |

**Table S5-16. Impact of the campaign on the change in vaccine hesitancy among the respondents of age 35 years and above**

|  | **Crude model** | | | **Adjusted model*** | | | **Weighted/adjusted model**** | | |
| --- | --- | --- | --- | --- | --- | --- | --- | --- | --- |
| **Variables** | **Beta** | **95% CI***^1^* | **p-value** | **Beta** | **95% CI***^1^* | **p-value** | **Beta** | **95% CI***^1^* | **p-value** |
| **Prepost** | 11 | 1.3, 21 | 0.029 | 13 | 2.6, 24 | 0.017 | 14 | 8.9, 19 | <0.001 |
| **Treatment** | 2.9 | -0.78, 6.5 | 0.127 | 3.4 | -0.55, 7.4 | 0.095 | 3.9 | -0.27, 8.0 | 0.067 |
| **Prepost*Treatment** | -11 | -21, -0.57 | 0.042 | -14 | -25, -2.4 | 0.021 | -15 | -21, -8.3 | <0.001 |
| * Model adjusted for gender, highest educational attainment, and occupation  ** Treatment and control group matched and adjusted by age group, ender, highest educational attainment, and occupation  *^1^* CI = Confidence Interval | | | | | | | | | |

**Table S5-17. Impact of the campaign on the change in vaccine confidence among the respondents of age 35 years and above**

|  | **Crude model** | | | **Adjusted model*** | | | **Weighted/adjusted model**** | | |
| --- | --- | --- | --- | --- | --- | --- | --- | --- | --- |
| **Variables** | **Beta** | **95% CI***^1^* | **p-value** | **Beta** | **95% CI***^1^* | **p-value** | **Beta** | **95% CI***^1^* | **p-value** |
| **Prepost** | 0.59 | -1.4, 2.6 | 0.567 | 0.54 | -1.5, 2.6 | 0.600 | 0.93 | -0.70, 2.6 | 0.264 |
| **Treatment** | -0.45 | -1.7, 0.77 | 0.471 | -0.42 | -1.6, 0.79 | 0.496 | -0.53 | -1.8, 0.78 | 0.427 |
| **Prepost*Treatment** | 0.43 | -1.8, 2.7 | 0.711 | 0.46 | -1.8, 2.7 | 0.691 | 0.15 | -1.7, 2.0 | 0.871 |
| * Model adjusted for gender, highest educational attainment, and occupation  ** Treatment and control group matched and adjusted by age group, ender, highest educational attainment, and occupation  *^1^* CI = Confidence Interval | | | | | | | | | |

**Table S5-18. Impact of the campaign on the change in % of fully vaccinated (including the one who has a confirmed appointment for the remaining doses) among the respondents of age 35 years and above**

|  | **Crude model** | | | **Adjusted model*** | | | **Weighted/adjusted model**** | | |
| --- | --- | --- | --- | --- | --- | --- | --- | --- | --- |
| **Variables** | **Odds ratio** | **95% CI***^1^* | **p-value** | **Odds ratio** | **95% CI***^1^* | **p-value** | **Odds ratio** | **95% CI***^1^* | **p-value** |
| **Prepost** | 4.35 | 1.11, 29.0 | 0.064 | 4.94 | 1.21, 33.7 | 0.048 | 7.24 | 1.35, 38.8 | 0.021 |
| **Treatment** | 0.60 | 0.33, 1.06 | 0.084 | 0.60 | 0.33, 1.09 | 0.097 | 0.57 | 0.31, 1.05 | 0.071 |
| **Prepost*Treatment** | 0.59 | 0.08, 2.61 | 0.526 | 0.53 | 0.07, 2.44 | 0.455 | 0.38 | 0.06, 2.29 | 0.300 |
| * Model adjusted for gender, highest educational attainment, and occupation  ** Treatment and control group matched and adjusted by age group, ender, highest educational attainment, and occupation  *^1^* CI = Confidence Interval | | | | | | | | | |

**References**

1. Stuart EA, Huskamp HA, Duckworth K, et al. Using propensity scores in difference-in-differences models to estimate the effects of a policy change. *Health Serv Outcomes Res Methodol.* 2014;14(4):166-182.
