# Supplementary material for: A pre-post evaluation study of a social media-based COVID-19 communication campaign to improve attitudes and behaviors toward COVID-19 vaccination in Tanzania
Source: PLoS One. 2024 May 6;19(5):e0300206. doi: 10.1371/journal.pone.0300206 (PMC11073716; doi:10.1371/journal.pone.0300206)
Supplement: Attachment — Submitted filename: Comments.docx [file pone.0300206.s007.docx]

**Ref: PONE-D-23-21202**

A pre-post evaluation study of a social media-based COVID-19 communication campaign to improve attitudes and behaviors toward COVID-19 vaccination in Tanzania

Thank you for giving me the opportunity to review this interesting manuscript. Congratulations to the authors for their diligent work. While the manuscript is interesting, I have some queries as outlined below.

General comments

- The manuscript is well written in general. I appreciate that the authors prepared the manuscript according to the STROBE reporting checklist.
- The Methods section requires more information to ensure replicability.
- The Discussion section needs to be improved and deeper international discussion of the subject is required.
- Description of ethics statement is required as the study involved human participants. Any ethics approval from the IRB/ERC/REC? Was informed consent taken electronically or written?

Abstract

- Add objective in the background of the abstract.
- Sample size 5804 differs with (3443+2362 = 5805) throughout the manuscript. Is that typo? Please check.

Introduction

- The Introduction section is well written, and the study gap is clearly described.

Methods

- Is there any official hashtag, such as #AfricaCOVIDChampions mentioned in the introduction, that has been employed in Tanzania for the COVID-19 communication campaign? If so, please describe it.
- How did you approach the respondents? A detailed description of the data collection procedure should be presented. Any inclusion and exclusion criteria? Whether the data collection was conducted through social media channels? What specific tools were utilized—such as chat bots or Google Forms?
- Why pre campaign respondents exposed to treatment category?
- More variable explanation is required. For example, the operation definition of fully vaccinated in this study.
- To mention as “Oxford COVID-19 Vaccine Hesitancy Scale” to be comparable to other studies.
- Regarding Vaccine Confidence Scale questions, did the authors adapt from an existing reference, and if so, provide the relevant citation? Or were they developed for this study?
- How did you select variables to be included in the model? Any model fitting?
- How did you handle missing data as one-third of the outcome data is missing?
- I appreciate the use of propensity score matching method in the analysis.
- Regarding analysis in Vaccine Hesitancy and Vaccine Confidence Scales, how did you handle scoring if the respondents answered ‘Don’t Know’?

Results

- I recommend maintaining consistent decimal usage for p-values throughout the manuscript.
- Figure 2 provides the same information as Tables 2-4 and suggests removing the figure.
- I suggest providing the frequencies and percentages for each item in the Vaccine Hesitancy and Vaccine Confidence scales as supporting information tables.

Discussion

- Discussion should be improved by following the format 1) Summary of the main findings 2) Compare with previous studies 3) Strength and weakness 4) Implications.
- While the authors have conscientiously highlighted various limitations of the study, it is equally important to incorporate a discussion on its notable strengths.
- While the authors have provided a discussion on vaccine hesitancy from line 353 to 371, I observed a very few discussions regarding vaccine confidence and uptake in the discussion section. I suggest a more in-depth international discussion encompassing all three outcomes.
