# Supplementary material for: A pre-post evaluation study of a social media-based COVID-19 communication campaign to improve attitudes and behaviors toward COVID-19 vaccination in Tanzania
Source: PLoS One. 2024 May 6;19(5):e0300206. doi: 10.1371/journal.pone.0300206 (PMC11073716; doi:10.1371/journal.pone.0300206)
Supplement: Attachment — Submitted filename: Responses to reviewers_final.docx [file pone.0300206.s009.docx]

Thanks very much for this round of comments. Please see our point-by-point responses below.

Reviewer #2: - Description of ethics statement is mandatory as the study involved human participants. Any ethics approval from the IRB/ERC/REC? Was informed consent taken electronically or written? etc...

🡪 As stated in lines 107 – 109, “This study was a secondary analysis of the pre-and post-campaign data collected by the Access Challenge (TAC), the implementing agency of the campaign, for their internal monitoring and evaluation purposes.” We received de-identified data from the implementing agency for the purpose of this research, and an Institutional Review Board (IRB) review was deemed not required by New York University’s IRB. We further clarified this information in lines 135-137 and provided an NYU internal form confirming this information as Supporting Information 2. Please also note that “Informed consent was sought from all respondents using an electronic survey form at the time of the original data collection by the implementing agency, and only consented individuals participated in the survey.” This is clarified in lines 170-172.

LINE 192-196 It is unclear whether you selected the variables to be included in the adjusted model based on previous literatures or used p-value from the crude analysis.

🡪 As stated in the lines 192-196, we used both literature review and crude statistical tests during the descriptive analysis to select the variables.
